# Rhesus Monkeys Have a Counting Ability and Can Count from One to Six

**DOI:** 10.3390/brainsci11081011

**Published:** 2021-07-30

**Authors:** Weiming Sun, Baoming Li, Chaolin Ma

**Affiliations:** 1 Center for Neuropsychiatric Disorders, Institute of Life Science, Nanchang University, Nanchang 330031, China; sunweiming@email.ncu.edu.cn (W.S.); bmli@ncu.edu.cn (B.L.); 2 School of Life Science, Nanchang University, Nanchang 330031, China

**Keywords:** counting, monkeys, numerical competence, animal cognition

## Abstract

Counting ability is one of the many aspects of animal cognition and has enjoyed great interest over the last couple of decades. The impetus for studying counting ability in nonhuman animals has likely come from more than a general interest in animal cognition, as the analysis of animal abilities amplifies our understanding of human cognition. In addition, a model animal with the ability to count could be used to replace human subjects in related studies. Here we designed a behavioral paradigm to train rhesus monkeys to count 1-to-6 visual patterns presented sequentially with long and irregular interpattern intervals on a touch screen. The monkeys were required to make a response to the sixth pattern exclusively, inhibiting response to any patterns appearing at other ordinal positions. All stimulus patterns were of the same size, color, location, and shape to prevent monkeys making the right choice due to non-number physical cues. In the long delay period, the monkey had to enumerate how many patterns had been presented sequentially and had to remember in which ordinal position the current pattern was located. Otherwise, it was impossible for them to know which pattern was the target one. The results show that all three monkeys learned to correctly choose the sixth pattern within 3 months. This study provides convincing behavioral evidence that rhesus monkeys may have the capacity to count.

## 1. Introduction

Numerical competence refers to the capacity of animals to recognize and name the cardinal numbers correlated with varying amounts of items and to order these numerals in the correct way [1]. Davis and Perusse [2] expressed numerical competence as relative numerousness judgment, subtilizing, estimation, and counting.

Relative numerousness judgment is the simplest form of numerical competence, represented by discrimination between two quantities [3]. Subtilizing was elaborated and defined as the “immediate, correct assignation of number words to small collections of perceptual items”, emphasizing that it is basically a perceptual process, rather than a cognitive one [4]. Estimation was suggested to be the perceptual process underlying subtilizing when it applied to larger arrays of items. Although estimation is considered to require considerable numerical sophistication, the assignment of a meaningful numerical tag to larger arrays of items may reflect only a perceptual process. A wide variety of animal species including birds [5,6], insects [7], rodents [8,9,10], dogs [11,12], fishes [13,14], and nonhuman primates [15,16,17,18] have shown some degree of numerical competence, as discussed above.

Counting could be considered a higher level of mathematical process in numerical competence. The ability to count and to use the number of objects or events as a cue is a quality that humans perform routinely and efficiently [19,20]. Many types of counting behavior have been described in monkeys. However, the degree to which nonverbal organisms possess this ability is more controversial. Matsuzawa reported that the chimpanzee Ai was able to report the number of objects presented to it by selecting the corresponding key from six keys marked with the Arabic numerals 1-to-6, suggesting that Ai had the ability to use the symbols of abstract numbers, or, in other words, that Ai had the ability to count. In addition, they found that Ai could generalize the Arabic numeral symbols to several other things [15]. Later, Murofushi et al. reported that Ai was successful in matching a series of dots to the corresponding Arabic numbers, despite the dots varying in color, size, form, and pattern [16]. Sawamura et al. [21] trained monkeys to do action A (push a joystick five times) five times, then to do action B (spin the joystick five times) five times, then to do action A five times again, and to repeat this cycle. Since the time of five consecutive movements varied randomly between 20 and 46 s, they ruled out timing or rhythmic strategies for problem solving. However, their design of the counting experiment is worth discussing. Their results showed that the activity of counting-related neurons was modulated not only by the number of times (1, 2, 3, 4, and/or 5), but also by the motions (push or spin). On the one hand, the number tags used in the experiment were specific movements, whose explicit codes may allow the monkeys to participate in counting with other strategies, such as how they perceived the amount of exercise. On the other hand, their experimental design did not satisfy the principle of order independence, nor did it satisfy the principle of abstraction. 

Counting ability is one of the many aspects of animal cognition, and has enjoyed great interest over the last couple of decades. The impetus for studying counting ability in nonhuman animals has likely come from more than a general interest in animal cognition, as the analysis of animal abilities amplifies our understanding of human cognition [22,23]. In addition, a model animal with the ability to count could also be used to replace human subjects in related studies, especially in those with invasive interventions. However, the degree to which organisms have the counting ability is of great controversy due to the present lack of convincing behavioral evidence that nonhuman animals can mentally and “truly” count as human beings do [24]. 

In the past, people generally did not believe that animals had the ability to count for the main reason that behavioral task designs were often flawed and could not well exclude the possibility of animals using other strategies. Gelman [25] and Gallistel [26] outlined that a robust definition of counting should include five different principles: (1) One to one correspondence: each component of a counted set must correspond to one single numeron; (2) Stable order: numerons must be ordered in a sequence that is reproducible every time; (3) Cardinality: the last numeron in a sequence also represents the total numerosity of the set; (4) Abstraction: counting applies to homogeneous and heterogeneous groups of objects of both physical and mental construction; and (5) Order irrelevance: the number in which the numerons correspond to each item is not important in the counting process. 

We designed a set of counting tasks, strictly following the principles outlined by Gelman and Gallistel [25,26], and trained three rhesus monkeys to count visual patterns presented sequentially with long and irregular delay periods. Moreover, we applied a transfer test task and further checked that rhesus monkeys have a counting ability and can count from one to six.

## 2. Materials and Methods

### 2.1. Subjects

Three Rhesus (*Macaca mulatta*) monkeys, including 1 female (Monkey #1, 5 years old) and 2 males (Monkeys #2 and #3, both 4 years old), were used for this experiment. The monkeys were housed individually in their cages (cage size: 80 cm × 80 cm × 90 cm), at a temperature of 25 ± 2 °C in a clean room. The monkeys were monitored daily by the researchers and the animal care staff, and on every second day a veterinarian checked their health and welfare conditions. The monkeys were cared for in accordance with the Guide for the Care and Use of Laboratory Animals issued by the National Institutes of Health, USA. Food and water were available ad libitum. 

### 2.2. Experimental Procedure

Each monkey was trained on a set of visually guided counting tasks after becoming familiar with the environment. The monkey was seated in a primate chair with one hand fixed, facing toward a computer touch screen. The screen was 30 cm away from the monkey chair and was within range of the monkey’s touch. 

A white square pattern (4.5 × 4.5 cm^2^) was displayed at the lower center of the screen, serving as a signal for a trial to start (starting signal). The maximum duration of the white pattern was 1.5 s. Immediately after the monkey touched the starting signal, one or multiple yellow square pattern(s) were sequentially presented in the center of the screen (4.5 × 4.5 cm^2^). The maximum duration of the yellow pattern was 0.8 s. The monkey was required to make a response to a target pattern that appeared at a given ordinal position, inhibiting any response to patterns appearing at other ordinal positions. Interpattern intervals (IPI) were widely randomized from 0.5 to 1.5 s so that the monkey could not use a timing strategy to solve the counting problem. All the target patterns presented were the same size, color, location, and shape. If the monkey made a correct response, a high-frequency tone (1000 Hz, 0.1 s) was presented and a piece of apple (reward) was given; if an incorrect response was made, a low-frequency tone (300 Hz, 0.1 s) was presented and no reward was given. The high- and low-frequency tones served as feedback signals to the monkey for its response choice (Video S1).

The monkeys were trained five days per week between 8:00 a.m. and 10:00 a.m. Since food and water were freely available to monkeys each day, the number of trials completed each day depended largely on their intrinsic motivation. Each monkey repeated 50–100 trials (70 trials on average) in a daily session. 

### 2.3. Experimental Counting Tasks

**Figure 1:**
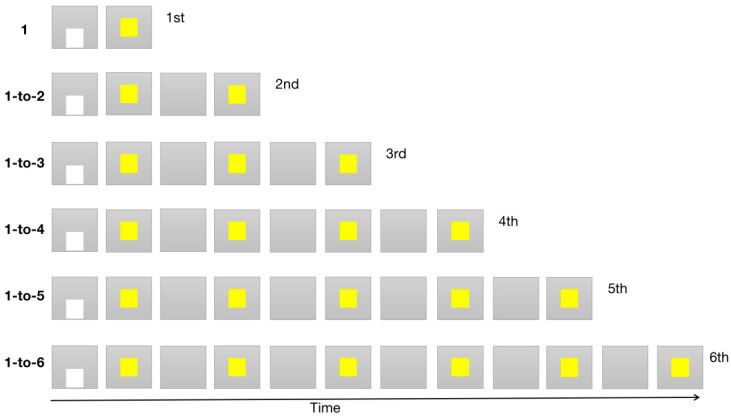
The 6 counting task protocols. Each task was started by touching the white square pattern (starting signal), then one or multiple yellow square pattern(s) sequentially appeared on the center of the screen, with 0.5–1.5 s interpattern intervals. In each task, the monkeys were required to make a response to a target pattern that appeared at a given ordinal position, inhibiting responses to patterns appearing at other ordinal positions. For example, they had to touch the 1st pattern in the 1-counting task, the 2nd pattern in the 1-to-2 counting task, the 3rd pattern in the 1-to-3 counting task, and so on. Ultimately, the monkeys learned to touch the 6th pattern in the 1-to-6 counting task.

The experiment consisted of 6 continuous tasks of increasing difficulty, as shown in Figure 1. Each monkey repeated 50–100 trials in a daily session. For the training process to proceed to the next counting task, the monkey had to have a correct response rate of at least 85% in 5 sequential daily sessions. The monkey was rewarded with a piece of apple for completing a task correctly.

For the first task (1-counting task), the 1st yellow pattern was the target, and the monkeys were required to make a response within 800 ms by touching the 1st yellow pattern. 

For the 1-to-2 counting task, the 2nd yellow pattern presented sequentially was the target, and the monkeys were required to touch the 2nd pattern and inhibit responses to the 1st yellow pattern. 

For the 1-to-3 counting task, the 3rd yellow pattern presented sequentially was the target, and the monkeys were required to touch the 3rd pattern and inhibit responses to the 1st and 2nd yellow patterns. 

For the 1-to-4 counting task, the 4th yellow pattern presented sequentially was the target, and the monkeys were required to touch the 4th pattern and inhibit responses to the 1st, 2nd, and 3rd yellow patterns. 

For the 1-to-5 counting task, the 5th yellow pattern presented sequentially was the target, and the monkeys were required to touch the 5th pattern, inhibiting any responses to patterns appearing at other ordinal positions. 

For the 1-to-6 counting task, the 6th yellow pattern presented sequentially was the target, and the monkeys were required to touch the 6th pattern, inhibiting any response to patterns appearing at other ordinal positions. 

### 2.4. Experimental Transfer Test Task

The ability of an animal to apply what it has learned from one situation to a new one is called transfer. An intramodal transfer reflecting the ability to count occurs if the monkeys are able to count correctly after switching to a new stimulus that has not been used in previous training [27]. A monkey’s counting transfer ability is a good indicator of whether the monkey is actually counting or not. Therefore, we designed a transfer test task to check if the counting stimuli can shift to heterogeneous groups with no impact on the monkey’s counting ability.

**Figure 2:**
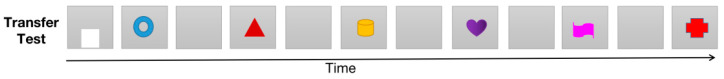
Transfer task protocol. After the monkey had learned the 1-to-6 task, all stimulus patterns at any ordinal position varied randomly in size, color, and shape. The monkeys were required to touch the 6th pattern, inhibiting any response to patterns appearing at other ordinal positions.

The transfer test was applied after the monkey had learned the 1-to-6 counting task. For the shift test task, the target was still the 6th pattern but all stimulus patterns at any ordinal position varied randomly in size, color, and shape. Interpattern intervals varied randomly between 0.5 and 1.5 s, and total trial duration varied randomly between 7.8 and 13.8 s. (Figure 2). 

### 2.5. Statistical Analysis

Statistical tests were all two-sided and *p* values ≤ 0.05 were considered to be statistically significant for each comparison. Comparisons of task training performance between monkeys and tasks were calculated using Pearson’s Chi-squared test. Analyses were conducted using IBM SPSS Statistics 20 (IBM SPSS, Turkey).

## 3. Results

### 3.1. Behavioral Performance of All Three Monkeys in the Experimental Counting Task

**Figure 3:**
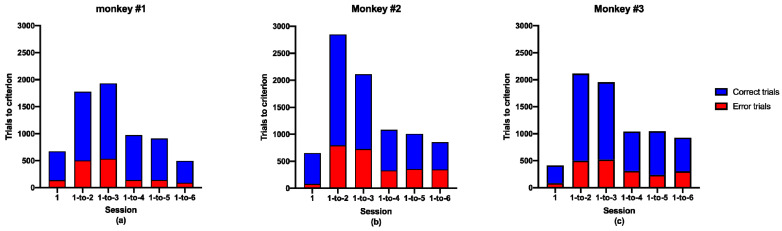
Counting task performance. (**a**) Training records of Monkey #1 in each of the tasks, including the total trials and errors before satisfying the test criterion; (**b**) Training records of Monkey #2; (**c**) Training records of Monkey #3.

A successful completion rate of 85% or more in five sequential daily sessions is a good criterion to establish when monkeys have learned a complex cognitive behavioral task [28]. Our results show that the three monkeys learned all six “counting tasks” within 3 months (Figure 3). 

Figure 3a shows the training records of Monkey #1 in all of the six counting tasks, including the total number of correct and erroneous trials before satisfying the test criterion. The 1-counting task is a very simple time-response task, requiring monkeys to touch the first pattern. Table 1 shows that the three monkeys all spent the least time on this stage. Monkey #1 completed the first counting task after 674 trials and made 135 errors. The 1-to-2 counting task required monkeys to touch the second pattern presented sequentially, inhibiting responses to the first yellow pattern (Figure 1). This is not a simple time-response task. Monkey #1 required 1785 trials and made 503 errors before she learned the task. The 1-to-3 counting task required monkeys to touch the third pattern presented sequentially, inhibiting responses to the first and second patterns (Figure 1). Monkey #1 required 1946 trials and made 53 errors before she learned the task. The 1-to-4 counting task required monkeys to touch the fourth pattern presented sequentially, inhibiting responses to the first, second, and third patterns (Figure 1). Interestingly, the number of trials and the errors to criterion for Monkey #1 decreased dramatically for this task. After that, she learned the 1-to-5 and 1-to-6 counting tasks quite quickly (*p* < 0.001, Table 1). It seemed that she gained some insight or changed her strategy to solve the issue after the fourth stage. 

The other two monkeys followed a similar trend to Monkey #1 (Figure 3b,c), but showed slight differences in every task. It seemed that Monkey #1 gained some insight or changed her strategy to solve the issue after the 1-to-4 stage, however, Monkey #2 and Monkey #3 showed similar behaviors after the 1-to-3 and the 1-to-2 stage, respectively (Table 1). They also varied in the number of trials, time spent, and accuracy rate, etc. This suggests that the three monkeys, while all able to learn counting tasks, may have different learning abilities and strategies. The performance and differences of the three monkeys at different stages of the counting task are detailed in Table 1 and the following Table 2. 

### 3.2. Behavioral Performance of All Three Monkeys in the Transfer Task

After learning the 1-to-6 counting task, we changed the signal from a yellow square to a random shape, size and color, and asked the monkeys to touch the sixth signal pattern given in a sequence. 

**Figure 4:**
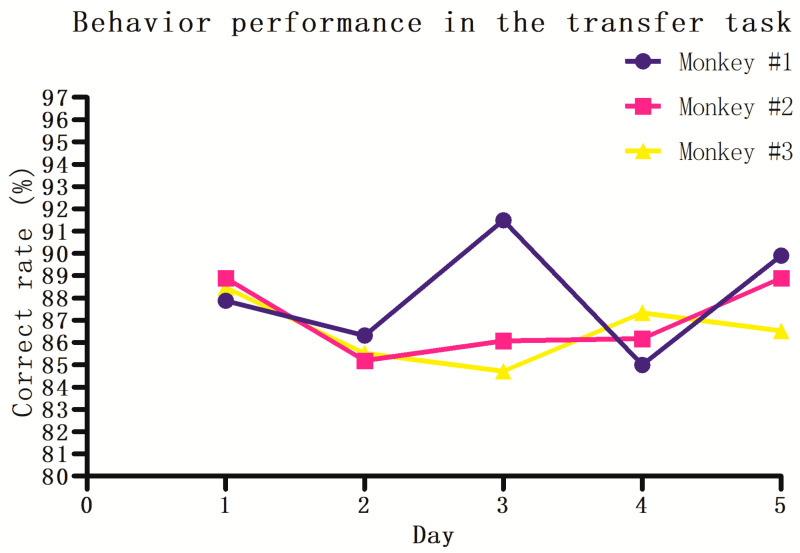
Behavioral performance of all three monkeys in the transfer task. Data shown are the correct rates from the first and the four subsequent daily sessions.

A successful completion rate of 85% or more in five sequential daily sessions was the criterion to establish that monkeys had learned the counting task in this study [28]. The results show that on the first test day, all three monkeys performed very well (>85%), and the performance of all three monkeys remained above 85% for five consecutive days (Figure 4). This further indicates that the monkeys did not rely on non-numerical factors such as the shape, size, or color of the signal itself to succeed in the counting task. This means that the three monkeys have a counting ability and can count from one to six. 

## 4. Discussion

Counting is the ability to execute quantitative assessments of objects and events and is one of the most important cognitive functions of human and animal brains. Data accumulated to date indicate that animals can recognize numerical symbols and use them in adaptive activities flexibly [20]. 

With the aim of providing a convincing behavioral demonstration that rhesus monkeys have a rudimentary ability to truly count, we designed a behavioral task—rigorously and carefully controlled to strictly follow the principles of Gelman [25] and Gallistel [26]—that required monkeys to respond correctly to sequential stimuli given by a computer. Our results show that all three monkeys learned the 1-to-6 counting task; they knew to make a response to the sixth pattern exclusively, inhibiting responses to any patterns appearing at other ordinal positions.

If the IPI were presented at constant intervals, the monkeys might have the ability to establish a relationship between the waiting time and 1-to-n pattern counting, and thus complete the trials by estimating time [28]. In that case, the detection function is not counting, but time estimation and continuous attention functions. As described in the experimental procedure, total trial duration in a 1-to-n trial = the white pattern duration × 1 + the yellow pattern duration × n + IPI × (n − 1). The maximum duration of the white pattern was 1.5 s. The maximum duration of the yellow pattern was 0.8 s. The IPI were randomized from 0.5 to 1.5 s. It was found that the duration of each trial was distributed randomly, so that the monkeys had difficulty guessing when to touch the screen correctly using time estimation strategies. In addition, the monkeys did not know which pattern was the last. In the long delay period, the monkeys had to enumerate how many successive patterns had been presented sequentially and remember which ordinal position the current pattern was in. Otherwise, it was impossible for them to know which pattern was the right target [29]. In addition, the size, color, location, and shape of the yellow pattern in every trial was exactly the same, meaning that the monkeys made correct choices independent of non-number physical parameters. It was impossible for the monkey to use a timing strategy to manipulate the task; it had to give the exact number of labels to each successive yellow box in order to make the correct response.

Insight is an advanced form of learning, and the higher the animal, the more developed it is. The insight process involves understanding the problem, thinking about the problem, and solving the problem [30]. An animal can think about the problem in terms of possible responses and predict the success rate of each attempt based on past experience. Problem solving often results from a combination of previous learning experiences [31]. From Figure 3, we found that monkeys learned the 1-to-4 counting task very quickly. Compared with the 1-to-2 and 1-to-3 tasks, the number of trials and the errors to criterion decreased dramatically. Subsequently, the three monkeys learned the 1-to-5 and 1-to-6 counting tasks relatively easily. Monkeys may apply different strategies in these larger number-counting tasks [32]. They may use interior code to enumerate how many patterns had been presented sequentially, instead of explicit code to tag stimulus patterns.

In the transfer task, the patterns at any ordinal position randomly varied in size, color, and shape. The target was still the sixth pattern presented sequentially, and the monkeys were required to make responses within 800 ms by touching the target after inhibiting any responses to patterns appearing at other ordinal positions. Other conditions were the same as in the 1-to-6 counting task, including interpattern intervals, widely randomized from 0.5 to 1.5 s, and the substantially delayed time of the sixth pattern presented, randomized from 7 to 13 s. We found that all three monkeys performed very well; the correct rates in the first and the four subsequent daily sessions were all above 85%. This suggests that the monkeys’ counting ability can shift between two or more types of patterns, indicating that the counting process of monkeys is rather abstract. At this point, we can say that our behavioral results confirm that monkeys are capable of counting, at least from one to six.

Counting is required for a large number of daily activities and is often implicated in more complex calculations and mathematical tasks [33]. In this respect, serial counting could be considered a basic ability in people’s daily lives. The development of numerical competence, including counting ability, is one of the higher cognitive functions of the human brain. However, clinical practice and experimental data accumulated to date suggest that the basic mechanism for sensing numbers has deep evolutionary roots and appeared before speech [20]. In addition, clinical observations of patients with lesions to the cerebral cortex, along with experiments on children of preverbal age and animals, have shown that there is a common biologically important and evolutionarily developed adaptive function associated with the perception of numbers of objects and events [33]. We hypothesized that the counting ability would have evolved in species that have evolved in socially complex societies, such as nonhuman primates.

## 5. Conclusions

So far, we may draw a conclusion that monkeys have a counting ability and can count from one to six.

However, this study had some limitations. First, the small sample size may have influenced the findings. Although we found that monkeys can count from one to six using the customized 1-to-6 task, we have not studied the maximum number to which monkeys can count (1-to-n). Moreover, whether the counting ability of monkeys is innate or learned through task training is another interesting research question. It is also worth studying and finding the brain regions related to counting function in monkeys. In follow-up research, we will try to address these problems by expanding the sample size and using a variety of neurobiological techniques.

## Figures and Tables

**Table 1:** Differences in counting task performance for different stages.

Monkey			Counting Task Session						Total	Pearson Chi-Square	df	*p*
			1	1-to-2	1-to-3	1-to-4	1-to-5	1-to-6				
#1	Correct	Frequency	536 _a_	1273 _b_	1396 _b_	834 _c_	770 _a,c_	409 _a,c_	5218	135.421 ^b^	5	0.000
		Expected Frequency	518.2	1371.5	1490.4	751.4	702.7	383.8	5218.0			
	Error	Frequency	135 _a_	503 _b_	534 _b_	139 _c_	140 _a,c_	88 _a,c_	1539			
		Expected Frequency	152.8	404.5	439.6	221.6	207.3	113.2	1539.0			
#2	Correct	Frequency	572 _a_	2055 _b_	1388 _c_	754 _b,c_	652 _c,d_	505 _d_	5926	179.805 ^c^	5	0.000
		Expected Frequency	450.9	1973.5	1463.7	749.5	697.5	590.9	5926.0			
	Error	Frequency	79 _a_	794 _b_	725 _c_	328 _b,c_	355 _c,d_	348 _d_	2629			
		Expected Frequency	200.1	875.5	649.3	332.5	309.5	262.1	2629.0			
#3	Correct	Frequency	327 _a_	1611 _a_	1434 _a,b_	727 _b,c_	810 _a_	624 _c_	5533	48.785 ^d^	5	0.000
		Expected Frequency	304.1	1562.1	1444.5	766.6	771.1	684.5	5533.0			
	Error	Frequency	84 _a_	500 _a_	518 _a,b_	309 _b,c_	232 _a_	301 _c_	1944			
		Expected Frequency	106.9	548.9	507.5	269.4	270.9	240.5	1944.0			
Total	Correct	Frequency	1435 _a_	4939 _b_	4218 _c_	2315 _b_	2232 _b_	1538 _c_	16,677	154.476 ^a^	5	0.000
		Expected Frequency	1268.2	4929.4	4387.1	2262.0	2165.4	1664.8	16,677.0			
	Error	Frequency	298 _a_	1797 _b_	1777 _c_	776 _b_	727 _b_	737 _c_	6112			
		Expected Frequency	464.8	1806.6	1607.9	829.0	793.6	610.2	6112.0			

Each subscript letter indicates a subset, and at the 0.05 level, the column proportions of these categories do not differ significantly from each other if they have the same letters. Each supscript letter indicates 0 cells (0%) have expected frequencies less than 5. ^a^ The minimum expected cell frequency is 464.79; ^b^ The minimum expected cell frequency is 113.20; ^c^ The minimum expected cell frequency is 200.06; ^d^ The minimum expected cell frequency is 106.86.

**Table 2:** Differences among the three monkeys in counting task performance.

Counting Task Session			Monkey			Total	Pearson Chi-Square	df	*p*
			#1	#2	#3				
1	Correct	Frequency	536 _a_	572 _b_	327 _a_	1435	18.771 ^b^	2	0.000
		Expected Frequency	555.6	539.1	340.3	1435.0			
	Error	Frequency	135 _a_	79 _b_	84 _a_	298			
		Expected Frequency	115.4	111.9	70.7	298.0			
1-to-2	Correct	Frequency	1273 _a_	2055 _a_	1611 _b_	4939	14.186 ^c^	2	0.001
		Expected Frequency	1302.2	2089.0	1547.8	4939.0			
	Error	Frequency	503 _a_	794 _a_	500 _b_	1797			
		Expected Frequency	473.8	760.0	563.2	1797.0			
1-to-3	Correct	Frequency	1396 _a_	1388 _b_	1434 _a_	4218	34.720 ^d^	2	0.000
		Expected Frequency	1357.9	1486.7	1373.4	4218.0			
	Error	Frequency	534 _a_	725 _b_	518 _a_	1777			
		Expected Frequency	572.1	626.3	578.6	1777.0			
1-to-4	Correct	Frequency	834 _a_	754 _b_	727 _b_	2315	88.473 ^e^	2	0.000
		Expected Frequency	728.7	810.4	775.9	2315.0			
	Error	Frequency	139 _a_	328 _b_	309 _b_	776			
		Expected Frequency	244.3	271.6	260.1	776.0			
1-to-5	Correct	Frequency	770 _a_	652 _b_	810 _c_	2232	106.431 ^f^	2	.000
		Expected Frequency	686.4	759.6	786.0	2232.0			
	Error	Frequency	140 _a_	355 _b_	232 _c_	727			
		Expected Frequency	223.6	247.4	256.0	727.0			
1-to-6	Correct	Frequency	409 _a_	505 _b_	624 _c_	1538	76.468 ^g^	2	0.000
		Expected Frequency	336.0	576.7	625.3	1538.0			
	Error	Frequency	88 _a_	348 _b_	301 _c_	737			
		Expected Frequency	161.0	276.3	299.7	737.0			
Total	Correct	Frequency	5218 _a_	5926 _b_	5533 _c_	16,677	125.512 ^a^	2	0.000
		Expected Frequency	4944.8	6260.6	5471.7	16,677.0			
	Error	Frequency	1539 _a_	2629 _b_	1944 _c_	6112			
		Expected Frequency	1812.2	2294.4	2005.3	6112.0			

Each subscript letter indicates a subset, and at the 0.05 level, the column proportions of these categories do not differ significantly from each other if they have the same letters. Each supscript letter indicates 0 cells (0%) have expected frequencies less than 5. ^a^ The minimum expected cell frequency is 1812.22; ^b^ The minimum expected cell frequency is 70.67; ^c^ The minimum expected cell frequency is 473.79; ^d^ The minimum expected cell frequency is 572.08; ^e^ The minimum expected cell frequency is 244.27; ^f^ The minimum expected cell frequency is 223.58; ^g^ The minimum expected cell frequency is 161.01.

## Data Availability

Not applicable.

## Supplementary Materials

The following are available online at https://www.mdpi.com/article/10.3390/brainsci11081011/s1, Video S1: 1-to-6 counting task.
